# Quantifying the contribution of established risk factors to cardiovascular mortality differences between Russia and Norway

**DOI:** 10.1038/s41598-020-77877-3

**Published:** 2020-11-27

**Authors:** Sergi Trias-Llimós, Lisa Pennells, Aage Tverdal, Alexander V. Kudryavtsev, Sofia Malyutina, Laila A. Hopstock, Olena Iakunchykova, Yuri Nikitin, Per Magnus, Stephen Kaptoge, Emanuele Di Angelantonio, David A. Leon

**Affiliations:** 1 Department of Non-Communicable Disease Epidemiology, Faculty of Epidemiology and Population Health, London School of Hygiene and Tropical Medicine, Keppel Street, London, WC1E 7HT UK; 2 Department of Public Health and Primary Care, University of Cambridge, Cambridge, UK; 3 Centre for Fertility and Health, Norwegian Insitute of Public Health, Oslo, Norway; 4 Central Scientific Research Laboratory, Northern State Medical University, Arkhangelsk, Russia; 5 Department of Community Medicine, Faculty of Health Sciences, UiT The Arctic University of Norway, Tromsø, Norway; 6 Research Institute of Internal and Preventive Medicine—Branch of IC&G, SB RAS, Novosibirsk, Russia; 7 Novosibirsk State Medical University, Ministry of Health of Russia, Novosibirsk, Russia; 8 International Laboratory for Population and Health, National Research University, Higher School of Economics, Moscow, Russian Federation

**Keywords:** Biomarkers, Risk factors

## Abstract

Surprisingly few attempts have been made to quantify the simultaneous contribution of well-established risk factors to CVD mortality differences between countries. We aimed to develop and critically appraise an approach to doing so, applying it to the substantial CVD mortality gap between Russia and Norway using survey data in three cities and mortality risks from the Emerging Risk Factor Collaboration. We estimated the absolute and relative differences in CVD mortality at ages 40–69 years between countries attributable to the risk factors, under the counterfactual that the age- and sex-specific risk factor profile in Russia was as in Norway, and vice-versa. Under the counterfactual that Russia had the Norwegian risk factor profile, the absolute age-standardized CVD mortality gap would decline by 33.3% (95% CI 25.1–40.1) among men and 22.1% (10.4–31.3) among women. In relative terms, the mortality rate ratio (Russia/Norway) would decline from 9–10 to 7–8. Under the counterfactual that Norway had the Russian risk factor profile, the mortality gap reduced less. Well-established CVD risk factors account for a third of the male and around a quarter of the female CVD mortality gap between Russia and Norway. However, these estimates are based on widely held epidemiological assumptions that deserve further scrutiny.

## Introduction

Large mortality differences exist between European countries, and particularly between Russia and Western European countries. Despite declines in mortality in Russia from the mid-2000s^1^, Russian life expectancy is still low compared to other countries. For example in 2014 it was 14.8 years shorter among men and 7.6 years shorter among women compared to Norway^2^. A key component of these differences is the very high mortality from cardiovascular disease (CVD) in Russia, particularly at working ages^1,3^.

Several attempts have been made to explain the elevated mortality rate in Russia including socioeconomic level, standard of medical care, and lifestyle factors^4–7^. It is acknowledged that behaviours such as smoking^8,9^ and alcohol intake^10–12^ and contextual factors such as gross domestic product (GDP) per capita^13^ are likely to contribute the elevated mortality rate in Russia.

CVD mortality is one of the most important contributors to mortality dynamics in Russia^14^, and it is much higher in Russia as compared to other European countries^3^, especially in the age group 40–69 years where it is 8–10 times higher compared to Norway^15^. This is likely to be partly explained by higher prevalence of established CVD risk factors. For example, prevalence of smoking remains very high among men^16^, and blood pressure levels are also high in both men and women^17^. In addition, the prevalence of hazardous alcohol drinking in Russia is one of the highest worldwide^18^.

There have been surprisingly few attempts to systematically examine the potential role of established risk factors on CVD mortality patterns between different countries^19^. The WHO MONICA Project^20,21^ is the most well-known attempt to address this issue using consistent methodology across countries. It aimed to explain changes in CVD mortality in relation to changes in incidence (risk factor levels) and case-fatality (treatment and medical services). It concluded that the established CVD risk factors were unlikely to explain the observed CVD mortality differences between the former communist countries and Western Europe between 1984 and 1986, although no attempt was made to quantify the extent to which the differences between countries could be explained^22^. More recently, the INTERHEART study quantified the contribution of risk factors to acute (non-fatal) myocardial infarction *within* each country, and concluded that established CVD risk factors explained a smaller fraction of total myocardial infarction incidence in Eastern Europe than in Western Europe between 1999 and 2003^23^. An earlier attempt to explain differences in CVD risk between Norway and Russia used data from 1987–1995 for Norway and from 1999 from Russia (Arkhangelsk)^24^. This study found that the population average myocardial infarction (fatal and non-fatal) risk score developed by Norway was lower for the Russian population than the two studies in Norway. They also found that the average Framingham risk score in Arkhangelsk was roughly the same as the Framingham average^24^.

In summary none of these previous studies actually quantified the fraction of differences in CVD mortality between populations that was accounted by established risk factors in either absolute or relative terms. Moreover, those that looked at Russia or Eastern Europe used data from the 1990s or earlier. In subsequent years CVD mortality in Russia has altered dramatically, most recently with substantial falls since 2005^1^.

The objectives of this paper are twofold. First, to quantify for the first time the joint contribution of four well-established CVD risk factors to the CVD (ischaemic heart disease -IHD- plus stroke) mortality gap between Russia and Norway at ages 40–69 years. Second, to identify and critically examine the assumptions underlying our approach many of which are common to epidemiological analysis and inference in general.

## Results

The means and prevalences of the four risk factors are shown in Table 1 by study. Age-specific summaries are presented in Appendix 3. SBP was appreciably higher in Know Your Heart (KYH, (Russia)) than in Tromsø 7 (Norway) for both men and women. Total cholesterol levels were similar in both studies for men and women separately. Smoking prevalence was 2–3 times higher in KYH for men, and only a slight difference of two percent points was observed for women. Diabetes prevalence was slightly higher in KYH for both men and women.

**Table 1:** Study specific age-standardized risk factor means (systolic blood pressure and total cholesterol) and prevalences (smoking and diabetes) with 95% confidence intervals.

	Data	SBP (mmHg)	Cholesterol (mmol)	Smoking (%)	Diabetes (%)
Men	Know your heart (Russia)	137.4 (136.5–138.4)	5.4 (5.3–5.5)	37.5% (35.0–39.9)	6.5% (5.3–7.7)
	Tromsø 7 (Norway)	130.8 (130.5–131.2)	5.5 (5.4–5.5)	13.7% (12.9–14.4)	5.2% (4.7–5.7)
	p-value for difference	< 0.001	0.025	< 0.001	0.055
Women	Know your heart (Russia)	128.6 (127.8–129.4)	5.7 (5.6–5.7)	17.2% (15.6–18.9)	8.2% (7.1–9.3)
	Tromsø 7 (Norway)	123.4 (123.1–123.8)	5.5 (5.5–5.6)	15.3% (14.5–16.0)	4.0% (3.6–4.4)
	p-value for difference	< 0.001	< 0.001	0.038	< 0.001

*SBP* systolic blood pressure.

*Age-specific estimates can be found in Appendix 3.

**Figure 1:**
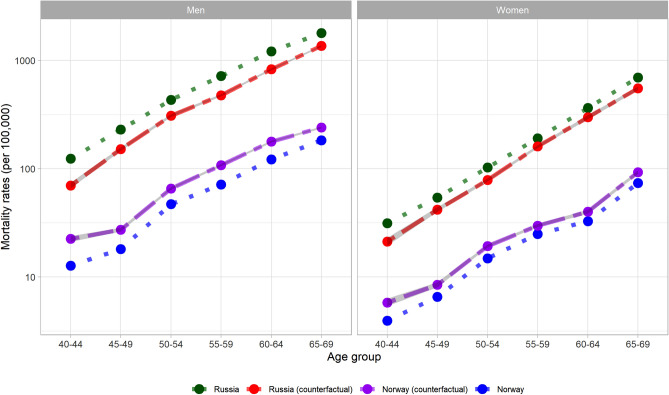
CVD mortality rates in Russia, Norway, Russia assuming the Norwegian risk factor profile (Russia counterfactual) and Norway assuming the Russian risk factor profile (Norway counterfactual).

CVD mortality rates for Russia and Norway and their alternate mortality rates when applying the counterfactual risk factor profile from the other country are depicted in Fig. 1 (and Appendix 4). In the counterfactual applying the Norwegian risk factor profile to Russia the estimated contributions of established risk factors to the excess age-specific CVD mortality in Russia than Norway ranged between 26.7% (ages 65–69) to 48.4% (ages 40–44) among men, and between 18.6% (ages 60–64) to 36.8% (ages 40–44) among women (Table 2, and Appendix 5 for IHD and stroke results). Overall, the absolute age-standardized CVD mortality gap would decline by 33.3% among men and 22.1% among women in this counter-factual scenario. In relative terms, the age-standardized mortality rate ratio (MRR, Russia/Norway) would decline from 10.0 to 7.0 among men and from 9.0 to 7.3 among women (Table 2). Conversely, in the counterfactual assuming Russian risk factor profile in Norway the observed results were in the same direction (identical MRR), but the impact was lower in absolute terms (ranging between 2.2 and 8.8%).

**Table 2:** Contribution of risk factors to the CVD mortality gap between Russia and Norway in absolute terms (%) and in relative terms (mortality risk ratios (MRR)).

	Age	MRR (Russia/Norway)	% Gap explained, Russia'	% gap explained, Norway'	Counterfactual MRR
Men	40–44	9.7	48.4 (44.4–52.1)	8.8 (7.6–10.1)	5.5 (5.2–5.8)
	45–49	12.7	36.9 (34.1–39.7)	4.4 (3.9–4.9)	8.4 (8.1–8.7)
	50–54	9.2	32.0 (29.5–34.3)	4.9 (4.3–5.4)	6.6 (6.4–6.8)
	55–59	10.1	37.4 (35.6–39.3)	5.6 (5.2–6.0)	6.7 (6.5–6.8)
	60–64	10.0	35.0 (33.4–36.8)	5.1 (4.8–5.5)	6.8 (6.7–7.0)
	65–69	9.8	26.7 (24.9–28.4)	3.6 (3.3–3.9)	7.5 (7.3–7.6)
	Age-standardized	10.0	33.3 (31.4–35.3)	4.8 (4.4–5.3)	7.0 (6.8–7.1)
Women	40–44	7.9	36.8 (30.4–43.0)	6.8 (5.2–8.7)	5.4 (5.0–5.8)
	45–49	8.3	25.9 (21.2–30.2)	4.0 (3.1–5.0)	6.4 (6.1–6.7)
	50–54	6.9	27.4 (24.7–30.1)	5.1 (4.5–5.8)	5.3 (5.2–5.5)
	55–59	7.7	18.6 (15.5–21.8)	2.9 (2.3–3.5)	6.4 (6.2–6.6)
	60–64	11.1	19.8 (15.9–23.6)	2.2 (1.7–2.7)	9.1 (8.7–9.5)
	65–69	9.4	22.8 (19.6–26.3)	3.0 (2.5–3.6)	7.5 (7.2–7.8)
	Age-standardized	9.0	22.1 (18.7–25.7)	3.1 (2.5–3.7)	7.3 (7.0–7.5)

95% uncertainty intervals denoted within brackets. Standard population: Russia 2014 (Human Mortality Database).

*Russia'* Russia assuming Norwegian risk factor prevalence, *Norway'* Norway assuming Russian risk factor prevalence.

## Discussion

This study used contemporary CVD risk models to quantify the risk factor contribution to the CVD (IHD plus stroke) mortality gap between two countries. To our knowledge this is the first attempt to quantify in relative and absolute terms the contribution of a combination of specific risk factors to differences in mortality from CVD between two countries. We found that well-established CVD risk factors (SBP, total cholesterol, smoking and diabetes) appear to account for about a third of the absolute CVD mortality gap between Russia and Norway among men, and around a quarter among women. The relatively low proportion of the gap explained by these risk factors is in line with other investigations^24^ including the MONICA study’s attempt to both explain international variation in CVD mortality cross-sectionally^22,25^ as well as trends over time in countries based on data on smoking, blood pressure and cholesterol^20,21^.

There are a number of potential explanations as to why these well-established risk factors only explain a small part of the CVD mortality gap. The most obvious of these is that there are other key determinants of cardiovascular mortality differences between countries that are in addition to smoking, blood pressure, cholesterol and diabetes. These include both other risk factors such as heavy and harmful alcohol consumption, exposure to particulate air pollution and differences in standard of primary and secondary prevention of CVD events and mortality through treatment.

Furthermore, there are two assumptions that deserve examination whose significance goes beyond this particular attempt to explain international differences. This is the extent to which the associations between the CVD risk factors and mortality are generalizable across populations and how far their combined influence on mortality risk really is best estimated on a multiplicative scale.

We did not include alcohol as one of the risk factors, despite the fact that harmful and episodic drinking is a risk factor and is prevalent in Russia where it has been associated with increased CVD risk^26,27^. The estimation of its impact to CVD mortality is complex and multidimensional^28^, especially in a context of high prevalence of binge drinking as is found in Russia^10,11^. Despite its importance we must recognize that alcohol is closely correlated with the conventional CVD risk factors included in our study such as smoking and blood pressure^29^, and the only attempt including alcohol in fatal atherosclerotic CVD risk prediction algorithms in Central and Eastern Europe failed to improve their performance^30^. Furthermore, as the relationship between alcohol and CVD in Russia is different than elsewhere and singularly complex^10^ we did not think it was reasonable to extrapolate from ERFC-derived alcohol-associated relative risks^28^.

Several other risk factors of potential importance were not included. For example, levels of salt intake in the diet may well vary between Russia and Norway. However, the assumption is that its effect on CVD mortality is largely going to be through its impact on blood pressure—which we did include, thus making this exclusion less problematic. Another relevant factor is particulate air pollution, which has also been linked to CVD events and mortality^31,32^, with levels of pollution being appreciably higher in Russia than in Norway^33^.

One potential explanation of the differences in risk that should be mentioned are genetic differences between the populations studied. It should be noted however, that the urban populations of Arkhangelsk and Novosibirsk are largely ethnic Russians, whose genetic ancestry overlaps substantially with that of Northern Europeans^34^. Nevertheless, a classic way to assess the potential contribution of genes vs environment is to undertake a study of migrants^35^. This would compare the mortality risks among Russians living in countries with much lower CVD risks. There are unfortunately very few such studies. In a study of migrants who moved (1990–2003) from the former Soviet Union to Israel Ott et al. found that their CVD mortality was one third of that of the population of the Russian Federation^36^. However, this study of recent first-generation migrants provides only some superficial insight into the importance or otherwise of environmental factors. Migrants in general are a special group of their country of origin, and will bring with them aspects of their lifestyles particularly if they migrate in adult life.

Overall, however, genetics is unlikely to explain much if any of the current differences in CVD mortality between Russia and Norway (and other Western European countries) for two other reasons. As already noted, CVD mortality in Russia has been falling very steeply since 2005, and the gap with Norway is closing. These major falls cannot be explained by genetics. Looking further back however in the 1960s CVD mortality in Russia was very similar to that in Western European countries such as the UK, Sweden and France in the 1960s^1^. It was only in the 1970s and 1980s that a large divergence arose.

One of the key factors that may contribute to CVD mortality differences is the effective treatment and medical prevention of CVD. There is evidence for example, that since 2010 there has been a reduction of in hospital mortality from myocardial infarction, most likely related to a very rapid expansion of percutaneous coronary intervention facilities across Russia^7,37^. Moreover, more effective and longer term control of blood pressure in the Norwegian population, and the irregular control of it in Russia^38^ may also be a relevant factor that is not reflected in the mean blood pressures per se. Not factoring in differences in standard of health care between Russia and Norway may account for some of the residual differences in mortality between Russia and Norway once the effects of the four risk factors are accounted for. At this more general level, Russia has a disadvantage as compared to Norway in other important socioeconomic determinants, for example in GDP per capita or in inequalities within each population. Socioeconomic differences and inequalities may explain some of the elevated risk factors profiles^39^, and it has been found that life expectancy in Russia is below that expected given its moderately high GDP^13^.

Beyond these potential explanations for the failure of the risk factors to explain the mortality gap, measurement error in exposures may contribute, including the use of single cross-sectional measures rather than longitudinal exposure profiles.

This study used measured (SBP and total cholesterol) and self-reported (diabetes and smoking) data collected in two cities in Russia and one in Norway. Efforts were made to accurately collect those data in comparable ways. A calibration study of biomarkers showed very small cross-laboratory differences in total cholesterol measurements between the two populations^40^, which made no substantial difference to our results. Furthermore, we need to acknowledge that our risk factor profiles come from three cities (two in Russia and one in Norway) and are not based on nationally representative samples. Nevertheless CVD mortality rates in Tromsø are rather similar to those for Norway as a whole, as are CVD mortality rates in Novosibirsk and Arkhangelsk similar to those in Russia as a whole^15^. The participation rate in KYH was 48%, although the educational profile as the participants was similar to that of the cities from which they came^15^. Moreover, the risk factor data is consistent with what previously observed, including smoking and blood pressure levels^41,42^. In the Tromsø Study the participation rate was 65% and risk factor prevalences we used from the Tromsø Study are comparable those from the HUNT Study in the county of Nord-Trøndelag, Norway^43,44^.

Ideally, instead of cross-sectional data, it would have been better to use life-course data from cohort studies within each country as longitudinal measures of exposures have been found to be more relevant for CVD mortality prediction for various behavioural factors^45^. However, directly comparable data of this type are not available. The fact that even nowadays important differences in risk factor profiles exist (e.g. higher blood pressure levels among Russians compared to Norwegians or three-fold higher smoking prevalence among Russian men compared to their Norwegian counterparts (see Table 1)) suggests that, despite narrowing the gap, the Russian CVD mortality disadvantage could persist for decades.

Our central information on the mortality risks associated with risk factor profiles were derived from 85 cohort studies from the ERFC. In using these estimates, we have assumed that the combined effect on mortality of different combinations of risk factors is multiplicative, with no interaction on the ratio scale. In other words, that the hazard ratios for one factor are independent of the level of every other risk factor, with the one exception of age. There is however some evidence from two different large pooled analyses that suggest the existence of quantitative and qualitative interactions between SBP and cholesterol on the risk of IHD and stroke^46,47^. If there are interactions on a multiplicative scale this would undermine the estimates we have made of the fraction of mortality accounted for by the four risk factors considered, although the magnitude and direction of bias are unclear. Testing the extent to which an interaction between risk factors could improve the estimates of joint effects on mortality should be a priority in large epidemiological studies and could aid improving comparative risk assessment estimates.

A second fundamental assumption we have made, which is also made throughout much of CVD epidemiology in general is that within a multiplicative framework, the association of each risk factor and mortality risk is the same across populations^48^. However, due to the paucity of large-scale follow-up studies of CVD risk factors in Russia relatively little work has been done to investigate how far CVD risk factors show a similar strength of association in Russia compared to other countries. What is known is that the association of alcohol on CVD mortality in Russia is not the same as elsewhere, a fact that has been incorporated into estimates of alcohol attributable mortality globally by WHO^18^.

The absolute difference in mortality explained differs according to the counterfactual used. This is because the effect on absolute mortality risk of applying any risk profile to a low baseline mortality group will be necessarily smaller than applying the same profile to a high mortality baseline group. In our case, the baseline group consist of individuals who are non-smokers, do not have diabetes and have optimal levels of blood pressure and cholesterol. Hence, the lack of symmetry between the two counterfactual estimates. Indeed, we show that the mortality rates in the “optimal” baseline group for Russia and Norway using the same methodology are appreciably lower than that in Russia (Appendix 6). These differences are largely going to reflect differences in levels of other risk factors as well as the impact of differences in standard of primary and secondary CVD prevention through the health sector.

The question as to which counterfactual best summarises the contribution of our four CVD risk factors to mortality differences is debateable. However, a priori the assumption is that the risk profile in Norway is more favourable as CVD mortality rates are so much lower than in Russia. Therefore, we focused the discussion based on the estimates provided by the counterfactual for which the Norwegian risk factor profile is applied to Russia.

In conclusion, our analyses suggest that the four well-established CVD risk factors analysed account for no more than a third of the CVD mortality gap between Russia and Norway. Our methodological extension using multivariable adjusted hazard ratios for multiple risk factors allowed us to provide, for the first time, estimates on the joint contribution of well-established CVD risk factors to CVD mortality differences between Russian and Norwegian contexts. Our careful consideration of the assumptions that need to be made in order to estimate the joint and simultaneous contribution of key risk factors to explaining mortality differences between populations illustrates the challenge that is faced in this area. In reality, the best estimates would be those based on large-scale cohort studies with comparable protocols. More broadly many of these assumptions are common to much epidemiological analysis and yet their validity on closer inspection has not been demonstrated. A more critical approach to these is a priority. These include the widely held assumptions that hazard ratios for CVD risk factors are generalizable across populations and that risks combine multiplicatively.

## Methods

### Data

We used three sources of information: (i) risk factor levels from recent population-based surveys carried out in two cities in Russia (Arkhangelsk and Novosibirsk, the Know Your Heart study, 2015–2018) and in one Norwegian city, Tromsø (Tromsø Study seventh survey, 2015–2016); (ii) mutually adjusted risk factor hazard ratios for CVD mortality from the WHO CVD risk prediction models based on pooled analyses of data from 85 cohort studies^48^; and (iii) contemporary observed national CVD mortality rates for Russia and Norway (2016).

Risk factor prevalence and levels were obtained from population-based cross-sectional surveys in Russia and Norway that jointly constitute the Heart to Heart collaboration^15^. For Russia we used information from the Know Your Heart (KYH) study (2015–2018), a cross-sectional survey carried out in two Russian cities (Arkhangelsk and Novosibirsk)^15^. For Norway we used data from the 7th wave of the Tromsø Study (Tromsø 7)^49,50^ (2015–2016). The risk factors included in the analysis were age (years), sex (male/female), systolic blood pressure (SBP, mmHg), total cholesterol (mmol/L), smoking (yes/no), and diabetes (yes/no). SBP and total cholesterol were measured, whereas diabetes and smoking where self-reported using similar approaches in the two studies (Appendix 1). Tromsø 7 had a participation rate of 66%, whereas KYH had a participation rate of 48%. The analysis included participants with complete data on all the relevant variables (3605 (86.8%) of 4153 participants in KYH, and 16,803 (95.2%) of 17,650 participants in Tromsø 7).

Data on the population age- and sex-specific mortality from ischaemic heart disease (IHD; ICD10: I20-I25) and stroke (I60–I69, G45) were retrieved from the Human Cause-of-Death database for Russia (2016) and from the WHO Mortality Database for Norway (2016).

### Analyses

Mutually adjusted sex-specific hazard ratios for mortality due to IHD and stroke were obtained from pooled analyses of population-based cohort data in the Emerging Risk Factors Collaboration (ERFC) adopting methods previously used in the derivation of the WHO CVD risk prediction models^48^. These models included the predictor variables: age, SBP, total cholesterol, smoking, diabetes and their interactions with age^48,51^ and hazard ratios were calculated using Cox proportional hazard regression model with duration (i.e. time from entry into the study) as the time scale. Appendix 2 details hazard ratios for the risk factor associations with IHD and stroke mortality. Deviation from the proportional hazards assumption was either minimal or non-existent, assessed by fitting models including time-varying covariates.

The estimation of the joint risk factor contribution to CVD mortality differences between Russia and Norway was done in several successive steps. First, for each individual in the Russian and Norwegian dataset we calculated their predicted mortality risks relative to the multidimensional baseline category using the WHO model coefficients. Secondly, for each sex, 5-year age group and CVD cause we calculated the mean values of these predicted risks in each study to estimate the predicted relative mortality hazard in Russia (RUS) and in Norway (NOR). The ratio of these predictive relative mortality hazards between studies (e.g. RUS/NOR) was taken as an estimate of the proportional change in CVD mortality that would occur if the risk factor distribution in the one population was counterfactually assumed to apply to the other. Thus, for example, for a particular sex, age group and CVD cause RUS/NOR is the proportional change in mortality hazard that would occur in Norway if this country had the counterfactual risk factor distribution seen in Russia for this particular age- and sex-group combination. The reciprocal (NOR /RUS) gives the equivalent proportional change in Russia that would occur if they had the counterfactual risk factor distribution of Norway. To generate uncertainty ranges we used the vector of point estimates and their variance–covariance matrix to make 1,000 random draws of the log hazard ratios from a multivariate normal distribution. We then used these drawn coefficient estimates to re-estimate the relative mortality hazards between studies, and considered the 2.5th and 97.5th centiles of the resulting distributions to be estimates of uncertainty ranges for the estimated counterfactual mortality rates ratios.

The absolute counterfactual cause-specific CVD mortality rate in each population (country, age, sex) was then estimated by multiplying the appropriate ratio by the corresponding observed mortality rate.

## Supplementary information

Supplementary Information
