# Supplementary material for: Quantifying the contribution of established risk factors to cardiovascular mortality differences between Russia and Norway
Source: Sci Rep. 2020 Nov 27;10:20796. doi: 10.1038/s41598-020-77877-3 (PMC7695740; doi:10.1038/s41598-020-77877-3)
Supplement: Supplementary file 1 — Supplementary Information [file 41598_2020_77877_MOESM1_ESM.docx]

**Quantifying the contribution of established risk factors to cardiovascular mortality differences between Russia and Norway**

Sergi Trias-Llimós^1*^, Lisa Pennells^2^, Aage Tverdal^3^, Alexander V. Kudryavtsev^4,5^, Sofia Malyutina^6,7^, Laila A. Hopstock^5^, Olena Iakunchykova^5^, Yuri Nikitin^6^, Per Magnus^3^, Stephen Kaptoge^2^, Emanuele Di Angelantonio^2^, David A. Leon^1,5^

1. Department of Non-communicable Disease Epidemiology, Faculty of Epidemiology and Population Health, London School of Hygiene & Tropical Medicine (United Kingdom)
2. Department of Public Health and Primary Care, University of Cambridge (United Kingdom)
3. Centre for Fertility and Health, Norwegian Insitute of Public Health, Oslo (Norway)
4. Central Scientific Research Laboratory, Northern State Medical University, Arkhangelsk (Russia)
5. Department of Community Medicine, Faculty of Health Sciences, UiT The Arctic University of Norway, Tromsø (Norway)
6. Research Institute of Internal and Preventive Medicine - Branch of IC&G SB RAS, Novosibirsk (Russia)
7. Novosibirsk State Medical University, Ministry of Health of Russia, Novosibirsk (Russia)

**Supplementary material**

**Appendix 1**. Comparison of questions about risk factors in Know Your Heart and Tromsø 7

|  | Systolic blood pressure (SBP) | Total cholesterol | Diabetes | Smoking |
| --- | --- | --- | --- | --- |
| KNOW YOUR HEART | Systolic blood pressure (mmHg) (3 measurements with an automated device with properly sized cuff after two minutes seated rest, mean of reading 2 and 3 were used in the analysis) | Total cholesterol measured in blood (mmol/L) | Have you ever been told by a doctor or nurse that you have diabetes mellitus? | Are you a current smoker? |
| TROMSO 7 | Systolic blood pressure (mmHg) (3 measurements with an automated device with properly sized cuff after two minutes seated rest, mean of reading 2 and 3 were used in the analysis), with variable cuff size | Total cholesterol measured in blood (mmol/L) | Do you have, or have you had diabetes? | Do you smoke daily? |

**Appendix 2**. Log hazard ratios, and standard errors for CVD, IHD, and Stroke mortality associations (WHO/ERFC)

|  |  | Men | | Women | |
| --- | --- | --- | --- | --- | --- |
|  |  | log HR | SE log HR | log HR | SE log HR |
| **CVD (IHD plus stroke) mortality** | |  |  |  |  |
|  | Age at baseline per 5 year | 0.5562 | 0.0171 | 0.7268 | 0.0259 |
|  | Total cholesterol per 1 mmol/L | 0.1514 | 0.0124 | 0.1095 | 0.0202 |
|  | Systolic blood pressure per 20 mmHg | 0.3785 | 0.0126 | 0.4069 | 0.0200 |
|  | History of diabetes | 0.8227 | 0.0497 | 1.1730 | 0.0817 |
|  | Current smoking | 0.6626 | 0.0319 | 0.9793 | 0.0568 |
|  | T. cholesterol interaction with age* | -0.0357 | 0.0061 | -0.0303 | 0.0092 |
|  | SBP interaction with age* | -0.0278 | 0.0066 | -0.0320 | 0.0094 |
|  | Diabetes interaction with age* | -0.0865 | 0.0241 | -0.1425 | 0.0366 |
|  | Smoking interaction with age* | -0.0885 | 0.0165 | -0.1157 | 0.0280 |
| **IHD mortality** | |  |  |  |  |
|  | Age at baseline per 5 year | 0.5296 | 0.0196 | 0.7444 | 0.0348 |
|  | Total cholesterol per 1 mmol/L | 0.1917 | 0.0136 | 0.1729 | 0.0230 |
|  | Systolic blood pressure per 20 mmHg | 0.3412 | 0.0145 | 0.3954 | 0.0251 |
|  | History of diabetes | 0.8826 | 0.0554 | 1.3665 | 0.0968 |
|  | Current smoking | 0.6905 | 0.0363 | 1.1003 | 0.0725 |
|  | T. cholesterol interaction with age* | -0.0302 | 0.0068 | -0.0309 | 0.0104 |
|  | SBP interaction with age* | -0.0286 | 0.0077 | -0.0299 | 0.0121 |
|  | Diabetes interaction with age* | -0.0875 | 0.2730 | -0.1605 | 0.0438 |
|  | Smoking interaction with age* | -0.0851 | 0.0190 | -0.1356 | 0.3570 |
| **Stroke mortality** | |  |  |  |  |
|  | Age at baseline per 5 year | 0.6706 | 0.0364 | 0.7015 | 0.3870 |
|  | Total cholesterol per 1 mmol/L | -0.0308 | 0.0310 | -0.0156 | 0.0373 |
|  | Systolic blood pressure per 20 mmHg | 0.5171 | 0.0262 | 0.4306 | 0.0332 |
|  | History of diabetes | 0.5897 | 0.1184 | 0.7790 | 0.1562 |
|  | Current smoking | 0.5455 | 0.0705 | 0.7811 | 0.0933 |
|  | T. cholesterol interaction with age* | -0.0071 | 0.0147 | -0.0131 | 0.0167 |
|  | SBP interaction with age* | -0.0537 | 0.0132 | -0.0377 | 0.0152 |
|  | Diabetes interaction with age* | -0.0409 | 0.0539 | -0.0703 | 0.0677 |
|  | Smoking interaction with age* | -0.0666 | 0.0352 | -0.0765 | 0.0458 |

* age implies age at baseline. Age was centred at 60 years, SBP at 120mmHg, and total cholesterol at 6mmol/L.

Hazard ratios were estimated using methods previously described (The Lancet Global Health 7(10), e1332-e1345).

**Appendix 3.** Risk factor levels by age, sex and study

| Data | Sex | Age | Systolic blood pressure (mmHg) | Cholesterol (mmol/L) | Smoking (%) | Diabetes (%) |
| --- | --- | --- | --- | --- | --- | --- |
| KYH (Russia) | Men | 40-44 | 131.8 (129.3-134.3) | 5.4 (5.2-5.5) | 37.8% (31.0-44.7) | 3.6% (1.0-6.3) |
|  |  | 45-49 | 132.5 (130.2-134.8) | 5.4 (5.2-5.5) | 38.5% (32.1-44.9) | 4.0% (1.4-6.5) |
|  |  | 50-54 | 135.7 (133.6-137.8) | 5.5 (5.4-5.6) | 41.7% (35.5-47.9) | 4.0% (1.6-6.5) |
|  |  | 55-59 | 139.7 (137.3-142.0) | 5.5 (5.3-5.6) | 41.0% (35.0-47.0) | 7.3% (4.1-10.4) |
|  |  | 60-64 | 142.0 (139.7-144.3) | 5.4 (5.3-5.5) | 35.5% (30.1-41.0) | 10.6% (7.1-14.1) |
|  |  | 65-69 | 145.4 (143.1-147.7) | 5.3 (5.1-5.4) | 28.1% (23.1-33.2) | 10.9% (7.4-14.5) |
|  | Women | 40-44 | 119.8 (118.0-121.6) | 5.1 (5.0-5.2) | 21.7% (17.1-26.2) | 1.6% (0.2-3.0) |
|  |  | 45-49 | 121.2 (119.6-122.9) | 5.5 (5.4-5.6) | 23.9% (19.1-28.7) | 2.0% (0.4-3.6) |
|  |  | 50-54 | 126.7 (124.9-128.6) | 5.8 (5.7-5.9) | 23.9% (19.2-28.6) | 3.1% (1.2-5.1) |
|  |  | 55-59 | 131.2 (129.2-133.3) | 5.9 (5.8-6.1) | 13.6% (10.0-17.1) | 9.0% (6.0-12.0) |
|  |  | 60-64 | 136.8 (134.8-138.8) | 5.8 (5.7-5.9) | 9.6% (6.6-12.6) | 16.6% (12.8-20.4) |
|  |  | 65-69 | 139.4 (137.5-141.4) | 5.8 (5.7-6.0) | 7.2% (4.7-9.7) | 20.8% (16.9-24.7) |
| Tromsø 7 (Norway) | Men | 40-44 | 126.6 (125.8-127.4) | 5.4 (5.4-5.5) | 10.4% (8.8-12.0) | 2.3% (1.5-3.0) |
|  |  | 45-49 | 126.9 (126.2-127.7) | 5.5 (5.4-5.5) | 13.7% (11.9-15.4) | 3.2% (2.3-4.1) |
|  |  | 50-54 | 129.5 (128.6-130.4) | 5.6 (5.6-5.7) | 14.9% (13.0-16.8) | 5.4% (4.2-6.6) |
|  |  | 55-59 | 132.4 (131.5-133.4) | 5.6 (5.5-5.6) | 15.9% (13.9-17.9) | 4.8% (3.7-6.0) |
|  |  | 60-64 | 134.5 (133.5-135.5) | 5.4 (5.4-5.5) | 14.8% (12.9-16.8) | 7.2% (5.7-8.6) |
|  |  | 65-69 | 137.1 (136.0-138.2) | 5.3 (5.2-5.3) | 12.4% (10.5-14.4) | 9.7% (7.9-11.4) |
|  | Women | 40-44 | 115.2 (114.6-115.9) | 4.9 (4.9-5.0) | 10.6% (9.1-12.1) | 2.5% (1.7-3.2) |
|  |  | 45-49 | 117.8 (117.0-118.5) | 5.2 (5.2-5.2) | 14.5% (12.8-16.2) | 3.4% (2.5-4.3) |
|  |  | 50-54 | 120.8 (120.0-121.6) | 5.6 (5.5-5.6) | 17.4% (15.5-19.2) | 3.4% (2.5-4.3) |
|  |  | 55-59 | 125.2 (124.3-126.2) | 5.9 (5.8-5.9) | 18.2% (16.2-20.2) | 3.8% (2.8-4.8) |
|  |  | 60-64 | 130.7 (129.6-131.7) | 5.9 (5.8-5.9) | 16.6% (14.6-18.6) | 5.3% (4.1-6.5) |
|  |  | 65-69 | 134.5 (133.3-135.7) | 5.9 (5.8-5.9) | 14.8% (12.8-16.8) | 6.4% (5.0-7.8) |

**Appendix 4.** CVD mortality rates (per 100,000) in Russia, Norway, Russia assuming the Norwegian risk factor profile (Russia counterfactual) and Norway assuming the Russian risk factor profile (Norway counterfactual)

|  |  | Russia | Russia (counterfactual) | Norway (counterfactual) | Norway |
| --- | --- | --- | --- | --- | --- |
| Men | 40-44 | 124 | 70 (66-74) | 22 (21-24) | 13 |
|  | 45-49 | 230 | 152 (146-158) | 27 (26-28) | 18 |
|  | 50-54 | 432 | 309 (300-319) | 66 (64-68) | 47 |
|  | 55-59 | 720 | 477 (465-488) | 108 (105-111) | 72 |
|  | 60-64 | 1,215 | 832 (813-850) | 178 (174-182) | 122 |
|  | 65-69 | 1,792 | 1,363 (1,334-1,391) | 240 (235-245) | 183 |
| Women | 40-44 | 31 | 21 (20-23) | 6 (5-6) | 4 |
|  | 45-49 | 54 | 42 (40-44) | 8 (8-9) | 7 |
|  | 50-54 | 103 | 79 (76-81) | 19 (19-20) | 15 |
|  | 55-59 | 191 | 161 (155-166) | 30 (29-31) | 25 |
|  | 60-64 | 365 | 299 (287-312) | 40 (38-42) | 33 |
|  | 65-69 | 697 | 554 (533-574) | 93 (89-96) | 74 |

**Appendix 5.** Contribution of risk factors to the ischemic heart disease (IHD) and stroke mortality gap between Russia and Norway in absolute terms (%) and in relative terms (mortality risk ratios (MRR))

| IHD | |  |  |  |  |
| --- | --- | --- | --- | --- | --- |
|  | Age | MRR (Russia/Norway) | % gap explained, Russia' (%) | % gap explained, Norway' (%) | counterfactual MRR |
| Men | 40-44 | 10.7 | 47.5 (42.3-52.0) | 7.8 (6.4-9.2) | 6.1 (5.6-6.6) |
|  | 45-49 | 11.3 | 36.5 (32.8-36.9) | 4.9 (4.2-5.5) | 7.5 (7.2-7.9) |
|  | 50-54 | 8.5 | 31.8 (28.8-34.5) | 5.2 (4.6-5.9) | 6.1 (5.9-6.3) |
|  | 55-59 | 8.8 | 37.9 (35.5-40.1) | 6.5 (5.9-7.1) | 5.8 (5.7-6.0) |
|  | 60-64 | 8.8 | 35.2 (33.1-37.4) | 5.8 (5.4-6.4) | 6.0 (5.9-6.2) |
|  | 65-69 | 9.1 | 26.2 (24.3-28.4) | 3.8 (3.4-4.2) | 6.9 (6.8-7.1) |
|  | Age-standardized | 9.0 | 33.4 (31.0-35.7) | 5.3 (4.8-5.9) | 6.3 (6.1-6.5) |
| Women | 40-44 | 14.3 | 35.7 (28.6-42.6) | 3.7 (2.7-4.9) | 9.5 (8.6-10.5) |
|  | 45-49 | 8.9 | 27.9 (22.4-33.3) | 4.2 (3.1-5.3) | 6.7 (6.3-7.1) |
|  | 50-54 | 9.8 | 27.7 (24.6-31.1) | 3.8 (3.2-4.4) | 7.4 (7.1-7.7) |
|  | 55-59 | 6.9 | 22.1 (18.2-26.0) | 3.9 (3.1-4.8) | 5.6 (5.4-5.9) |
|  | 60-64 | 9.7 | 21.4 (17.1-26.4) | 2.7 (2.1-3.6) | 7.8 (7.4-8.2) |
|  | 65-69 | 10.0 | 26.0 (22.3-30.6) | 3.4 (2.8-4.2) | 7.6 (7.2-8.0) |
|  | Age-standardized | 9.1 | 24.4 (20.4-28.9) | 3.4 (2.7-4.2) | 7.2 (6.8-7.5) |
|  |  |  |  |  |  |
| Stroke | |  |  |  |  |
|  | Age | MRR (Russia/Norway) | % gap explained, Russia' (%) | % gap explained, Norway' (%) | counterfactual MRR |
| Men | 40-44 | 8.1 | 50.2 (39.0-59.5) | 11.1 (7.3-15.3) | 4.5 (3.9-5.3) |
|  | 45-49 | 18.6 | 39.8 (32.8-45.9) | 3.4 (2.5-4.3) | 11.6 (10.6-12.9) |
|  | 50-54 | 12.1 | 34.2 (28.4-39.5) | 4.1 (3.2-5.1) | 8.3 (7.7-9.0) |
|  | 55-59 | 16.5 | 36.3 (32.0-40.1) | 3.3 (2.8-3.9) | 10.9 (10.3-11.5) |
|  | 60-64 | 15.4 | 35.6 (31.8-39.1) | 3.5 (2.9-4.0) | 10.3 (9.8-10.8) |
|  | 65-69 | 12.0 | 27.9 (25.0-30.8) | 3.1 (2.7-3.6) | 8.9 (8.6-9.3) |
|  | Age-standardized | 13.6 | 34.0 (29.7-37.9) | 3.7 (3.0-4.4) | 9.3 (8.9-9.9) |
| Women | 40-44 | 5.4 | 36.8 (24.4-48.2) | 9.7 (5.6-14.7) | 3.8 (3.3-4.3) |
|  | 45-49 | 7.7 | 20.1 (12.2-28.1) | 3.2 (1.8-4.9) | 6.3 (5.8-6.9) |
|  | 50-54 | 5.0 | 26.2 (20.7-31.4) | 6.6 (4.9-8.3) | 4.0 (3.8-4.2) |
|  | 55-59 | 9.3 | 13.4 (9.1-17.8) | 1.6 (1.1-2.3) | 8.2 (7.8-8.6) |
|  | 60-64 | 15.1 | 16.3 (10.4-22.3) | 1.3 (0.8-1.9) | 12.8 (12.0-13.6) |
|  | 65-69 | 8.7 | 17.9 (12.7-23.2) | 2.4 (1.6-3.4) | 7.3 (6.9-7.7) |
|  | Age-standardized | 8.9 | 18.2 (12.6-23.8) | 2.6 (1.8-3.7) | 7.4 (7.0-7.9) |

Russia' = Russia assuming Norwegian risk factor prevalence

Norway' = Norway assuming Russian risk factor prevalence

**Appendix 6.** Comparison of observed CVD mortality and estimated counterfactual mortality for the “optimal” baseline groups in Russia and Norway*.


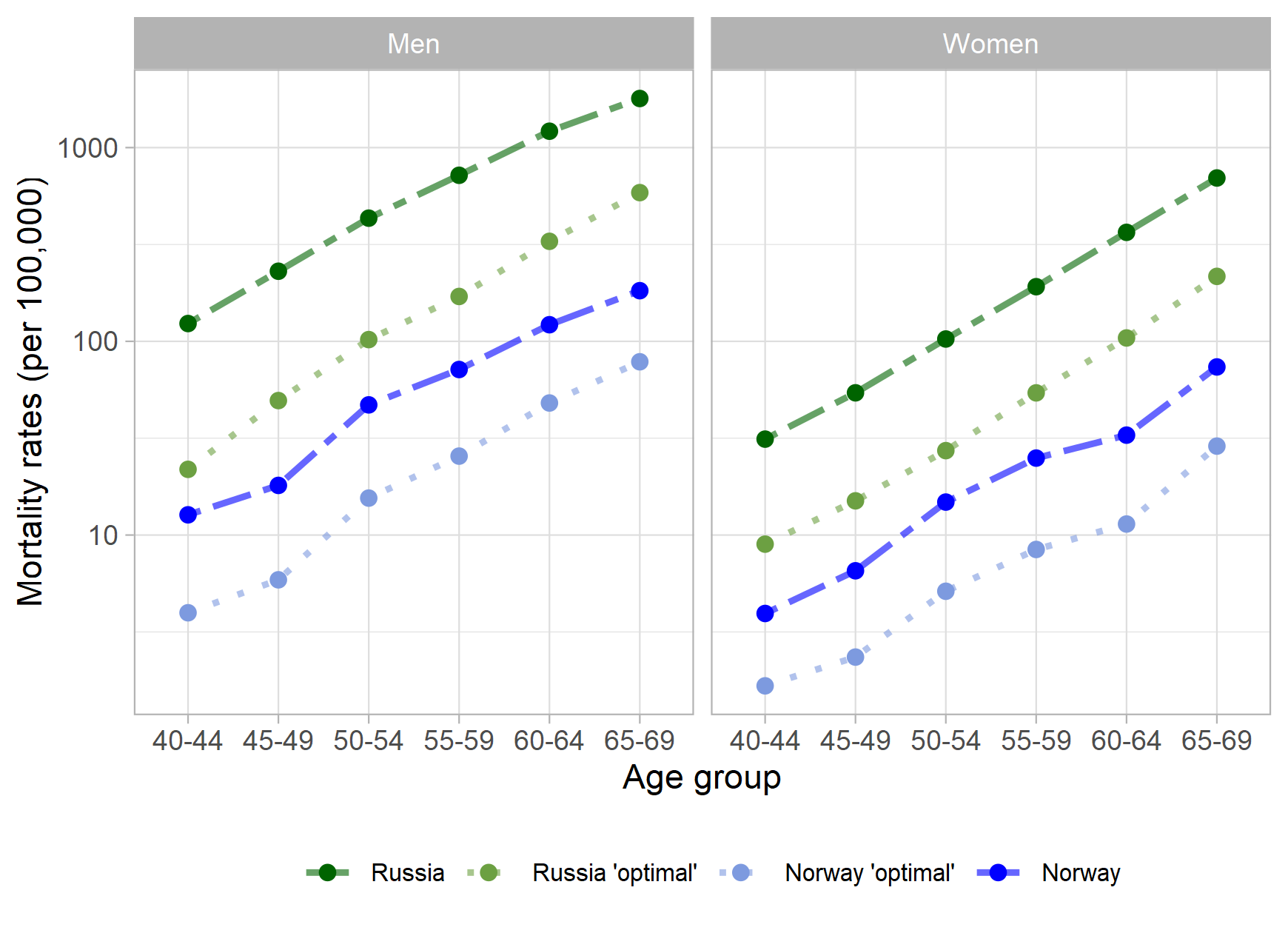


* “Optimal” baseline group was defined as having systolic blood pressure levels of 112.5 mmHg, total cholesterol levels of 3.9 mmol/L and being non-smoker and non diabetic.
